# Antimicrobial stewardship in private pharmacies in Wakiso district, Uganda: a qualitative study

**DOI:** 10.1186/s40545-023-00659-5

**Published:** 2023-11-17

**Authors:** David Musoke, Grace Biyinzika Lubega, Mimi Salome Gbadesire, Stephanie Boateng, Belinda Twesigye, Jagdeep Gheer, Betty Nakachwa, Michael Obeng Brown, Claire Brandish, Jody Winter, Bee Yean Ng, Kate Russell-Hobbs, Linda Gibson

**Affiliations:** 1 Department of Disease Control and Environmental Health, School of Public Health, College of Health Sciences, Makerere University, P. O. Box 7072, Kampala, Uganda; 2 Institute of Health and Allied Professions, School of Social Sciences, Nottingham Trent University, Nottingham, NG1 4FQ UK; 3 Medicines Optimisation Team, Buckinghamshire, Oxfordshire and Berkshire West Integrated Care Board Trust Offices, Amersham Hospital, Amersham, HP7 0JD UK; 4 Pharmacy Department, Buckinghamshire Healthcare NHS Trust, Aylesbury, HP21 8AL UK; 5 Department of Biosciences, School of Science and Technology, Nottingham Trent University, Nottingham, NG11 8NS UK; 6 Department of Pharmacy, Oxford University Hospitals NHS Foundation Trust, Oxford, OX3 9DU UK

**Keywords:** Antimicrobial resistance, Antimicrobial stewardship, Private pharmacies, Uganda

## Abstract

**Background:**

Private pharmacies are the first point of contact for the public regarding acquisition of medicines and other pharmaceuticals in many low- and middle-income countries including Uganda. Most antimicrobial stewardship (AMS) programmes in Uganda have targeted pharmacies in public health facilities, with little known about private pharmacies. This study explored knowledge and practices related to AMS in private pharmacies in Wakiso district, central Uganda.

**Methods:**

This was a qualitative study that involved 31 in-depth interviews to explore AMS among retail private pharmacy staff including pharmacists, pharmacy technicians/dispensers, and nurses. Participants were asked about antimicrobial resistance (AMR) and AMS practices at their pharmacy. The audio-recorded interviews were transcribed verbatim and imported to NVivo 2020 (QSR International) for thematic analysis.

**Results:**

Five major themes emerged from the study: commonly sold antimicrobials; knowledge on AMR and AMS; potential contributors to AMR; practices related to AMS; and challenges to AMS. The commonly sold antimicrobials in the pharmacies with or without prescriptions were oral azithromycin, Ampiclox^®^ (ampicillin and cloxacillin), amoxicillin, ciprofloxacin, Septrin^®^ (co-trimoxazole), metronidazole, Flucamox^®^ (amoxicillin and flucloxacillin), Augmentin^®^ (amoxicillin and clavulanic acid), cephalexin, doxycycline, and chloramphenicol. Participants had heard about AMR but not AMS, although only a few correctly defined AMR. Lack of knowledge among health workers and local communities; the overuse, misuse, and abuse of antimicrobials such as non-adherence to dosage; self-medication; and purchase of drugs without prescription were identified as potential accelerators to the emergence of AMR. Current practices related to AMS in private pharmacies were limited to meetings, antimicrobial dispensing, providing client advice, record keeping, and monitoring of drugs. Cost of healthcare, client satisfaction and retention, outdated guidelines, and the business orientation of pharmacies were the main challenges related to AMS.

**Conclusion:**

There was poor knowledge of AMR and AMS, and limited AMS practices in private pharmacies. Private pharmacies have the potential to contribute to Uganda’s fight against AMR if motivated and equipped with adequate knowledge to enhance their practices related to AMS.

## Introduction

In recent years, antimicrobials have become increasingly ineffective [1, 2] and as a result, antimicrobial resistance (AMR) is now classified among the top 10 threats to global health [3, 4]. AMR brings concerns regarding human and animal health, with escalating concerns of an approaching post-antibiotic era [1, 2, 5]. The implications of AMR negatively affect health systems globally with higher morbidity, mortality, and treatment costs. These effects are predicted to escalate, with the highest burden being evident in low- and middle-income countries (LMICs) such as Uganda [6–8]. Given that AMR can potentially affect anyone irrespective of their social and demographic characteristics, urgent attention and interventions are needed to curb the spread of this global public health challenge [9].

Antimicrobial stewardship (AMS) is a healthcare-system-wide approach to promoting appropriate use of antimicrobials in order to preserve their effectiveness. It entails appropriate antimicrobial prescribing interventions and strategies aimed at decelerating the emergence of AMR and preventing its unintended consequences [6, 10]. AMS programmes aim to ensure the efficacy of the available antimicrobials through dispensing and usage of appropriately prescribed medicines, following recommended dosages and durations, while minimizing their side effects and emergence of resistant pathogen strains [11]. Many players are crucial for successful human health AMS programmes including patients, health workers, pharmacists, and dispensers [12–14].

In Uganda, the Department of Pharmaceuticals and Natural medicines in the Ministry of Health (MOH) is tasked with overseeing policy development, implementation, and coordination of pharmaceutical activities in the country [15]. With support from the National Medical Stores, MOH is actively involved in monitoring rational use, as well as quantification and harmonization of the supply chain of all pharmaceutical and related commodities in the country [16]. The medicines health supply chain system in the country is made up of both public and private entities. All facilities are regulated by the National Drug Authority (NDA) which is also responsible for their annual issuance of licenses [15]. The private sector comprises health facilities including private pharmacies, drug shops, clinics, and hospitals [17, 18]. Most private health facilities procure their pharmaceutical products from a network of private-for-profit entities depending on the services offered and their logistical purchasing power [17].

Private pharmacies, also known as community pharmacies, are usually the first point of contact for the public when accessing medicines in many LMIC settings including Uganda [19, 20]. These pharmacies provide a range of medicines such as antibiotics, antimalarials, anti-fungals, other prescription drugs for different illnesses, and cosmetic products. Some of the common antibiotics dispensed in private pharmacies include oral macrolides, penicillins, nitroimidazole, first generation cephalosporins, tetracyclines, amphenicol, and sulfamethoxazole–trimethoprim [21, 22]. In addition, pharmacies are a point of contact for healthcare-related advice and can refer patients to other more appropriate facilities if necessary [23]. As such, private pharmacies play an important role in the supply of antimicrobials for infectious diseases in communities and triaging patients to the correct care setting. However, many studies have shown that private pharmacy staff are involved in improper prescribing practices, many of which are drivers of AMR [24, 25]. Some of the practices in private pharmacies include selling antimicrobials without prescriptions [26, 27], undersupply of antimicrobials despite the duration stipulated in prescription, and polypharmacy [28]. Other studies have highlighted gaps in knowledge on AMR and AMS among private pharmacy workers [28]. Furthermore, some private pharmacies are seen to be more interested in sales and profits, while others give in due to pressure and customer demands [29, 30]. Whereas policy regulation within the government pharmacies is more strict in Uganda, this is less so in private pharmacies. As a result, many of the policy guidelines are underlooked and not enforced, resulting in unregulated and improper use of antimicrobials in many privately owned pharmacies [31].

The World Health Organization (WHO) has advocated for more monitoring and awareness interventions among health practitioners to formulate robust and effective measures in tackling AMR [32]. In Uganda, a number of initiatives have been implemented to promote AMS in health facilities, with a particular focus on the pharmacy department [33–35]. These initiatives include commemoration of the World AMR Awareness Week, AMS workshops, as well as Infection Prevention and Control (IPC) strategies. However, many of these initiatives have targeted pharmacists in public health facilities, and private pharmacies have hardly been engaged. Indeed, the current evidence on AMS in Uganda is largely from public health facilities [33–35]. Private health facilities including pharmacies have a key role to play in offering consistent and informed advice and practices in relation to antimicrobial use to slow the development of AMR. Therefore, this study explored knowledge and practices related to AMS in private pharmacies in Wakiso district, Uganda in 2022.

## Methods

### Study design and participants

This was a qualitative study, conducted in 2022, that used in-depth interviews (IDIs) to explore knowledge and practices related to AMS among private pharmacies in Wakiso district, Uganda. A total of 31 IDIs were conducted among retail private pharmacy staff. From each pharmacy, at least one staff member was purposively selected based on their involvement in dispensing antimicrobials, with 3 pharmacies providing 2 participants. The cadre of the staff members interviewed and other socio-demographic characteristics were determined during the IDIs.

### Study area and setting

The study was conducted in Entebbe municipality and surrounding areas in Wakiso district, central Uganda. Entebbe municipality was chosen as the study site because of planned activities in the area as part of the Commonwealth Partnerships for Antimicrobial Stewardship (CwPAMS) scheme [36]. From prior workshops on AMS among health workers in the area, a concern was raised about how private pharmacies could be dispensing antimicrobials to clients without prescriptions [37]. This emerging issue particularly motivated the researchers to conduct the study. Entebbe municipality is situated in Wakiso district to the South West of Kampala, the capital city of Uganda. The municipality is located on one of Lake Victoria’s peninsulas of approximately 56.2 square kilometres. It is divided into two divisions A and B, with 24 zones. According to the last national population census, the municipality had a total population of 69,430 [38]. Entebbe municipality had approximately 30 pharmacies for both wholesale and retail among which 25 were for retail [39]. Pharmacies are operated by a range of health workers such as clinical officers, midwives, nurses, pharmacists, pharmacy technicians, and nursing assistants.

### Sampling and data collection

Initially, a list of 25 retail pharmacies in Entebbe including their physical address and contacts was obtained from the NDA website [39] and cross-checked with the database of the Pharmaceutical Society of Uganda (PSU). With the help of Google maps and a local community member, two Research Assistants (RAs) were able to locate the listed pharmacies. However, not all the listed pharmacies participated in the study as some had outdated contact details, incorrect locations, or closed businesses. In addition, 15 pharmacies declined to take part in the study due to various reasons hence 10 pharmacies were involved from Entebbe Municipality. These reasons included: being busy with clients; only one staff being available at the time; fear of supervisors finding their employees off duty while participating in the study; fear that the research team was from NDA, the national regulatory body; while others were not interested to take part. Only retail pharmacies were included in the study since wholesale pharmacies did not primarily dispense drugs to individual clients but rather sold drugs to retail pharmacies and drug shops. Pharmacies were selected consecutively for their participation in the study. Indeed, the RAs selected pharmacies as they came across them, based on their availability and willingness to participate in the study. In order to reach data saturation, 18 pharmacies in Katabi Town Council that neighbours Entebbe Municipality were involved in the study.

Two RAs with bachelors degrees in health sciences and prior experience in the private pharmacy setting carried out data collection under the supervision of the researchers. The RAs underwent training on data collection procedures before actual data collection. The IDI guide used to collect data was developed by the researchers who had expertise in AMR/AMS with professional backgrounds in various fields including pharmacy, public health, and microbiology. Validation of the tool was done by pharmacists from both Uganda and the UK. The guide had two main sections, which had questions exploring the structures and practices on AMS among private pharmacies. The first section had background questions on the staff’s role at the pharmacy, working conditions, and clients that they served. The second section was on participants’ views on AMR and AMS practices within the pharmacy. Before data collection, the IDI guide was piloted among pharmacies that were not within the study area and a few changes were made. The IDIs, conducted in English and audio recorded, lasted approximately 60 min and were held at the convenience of the staff found in the pharmacy. Each IDI was conducted by one of the RAs.

### Data management and analysis

The audio recordings from the IDIs were transcribed verbatim and proofread by the RAs to ensure that they were accurate. The generated transcripts were then imported into NVivo 2020 (QSR International) where data analysis was done by researchers experienced in qualitative research. Initially, the researchers familiarized themselves with the data through repetitive reading of the transcripts. Thereafter, generation of initial codes was undertaken by each of the researchers (MSG, SAB, GBL and JG). From the codes, the 4 researchers generated sub-themes and later themes that were reviewed in detail during two meetings. Where the researchers had disagreements regarding the codes and themes, a consensus was reached. At this step, some of the developed codes and themes were dropped, while new ones were generated. Throughout the data analysis process, the researchers referred to the steps of analytic rigour, credibility, transferability, dependability, and confirmability. In addition, notes taken during data collection supported contextual analysis. The initial analysis was performed by MSG, SAB, GBL and JG, and this was later cross-checked to facilitate the development of themes by GBL and DM. To support our analysis, selected quotes from the transcripts are provided with the findings.

### Ethical considerations

The study obtained ethical approval from Makerere University College of Health Sciences, School of Health Sciences Research and Ethics Committee (2019–-051) and was registered at the Uganda National Council for Science and Technology (HS 2711). Written informed consent was obtained from all the participants, and their participation was voluntary. Anonymity was ensured since the participants’ identifying information including their names was not obtained during data collection. Data from the study were only accessed by the research team to maintain confidentiality and was not used for any other purpose.

## Results

A total of 31 IDIs from 28 pharmacies were conducted in the study. Ten pharmacies with 12 participants from Entebbe Municipality, and 18 pharmacies with 19 participants from Katabi Town Council participated in the study. The majority of participants 61.3% (19/31) were female, with a mean age of 28.7 years and a standard deviation of 6.1. The majority of participants 58.1% (18/31) had a diploma, with the most common cadre being pharmacy technicians 29.0% (9/31). The majority of participants’ 80.6% (25/31) role was to dispense drugs. Nearly half of the pharmacies 48.4% (15/31) were solely owned by persons with a medical background, and only 19.4% (6/31) of the pharmacies dispensed antimicrobials for animal use. Only 25.8% of the participants (8/31) had previous training on AMS, while 67.7% (21/31) had been employed for 7 or less years (Table 1).

**Table 1:** Participants’ socio-demographic characteristics

Socio-demographic characteristics	Frequency (*n* = 31)	Percentage (%)
Gender		
Female	19	61.3
Male	12	38.7
Highest level of education		
Bachelors degree	1	3.2
Diploma	18	58.1
Certificate	12	38.7
Age (years)		
22–35	28	90.3
36–48	3	9.7
Cadre		
Clinical officer	3	9.7
Midwife	3	9.7
Nurse	6	19.4
Nursing assistant	5	16.1
Pharmacist	1	3.2
Enrolled nurse	2	6.5
Pharmacy technician	9	29.0
Psychiatric nurse	1	3.2
Social scientist	1	3.2
Participants’ role		
Dispenser	25	80.6
Manager	1	3.2
Prescriber	2	6.5
Supervisor	3	9.7
Ownership of pharmacy		
Group (medical)	7	22.6
Group (non-medical)	4	13.0
Sole ownership (medical)	15	48.4
Sole ownership (non-medical)	5	16.0
Years of employment		
< 1	10	32.3
1–7	21	67.7
Estimated number of clients per day		
1–50	25	80.6
50–100	6	19.4
Sale of antimicrobials for animal use		
No	25	80.6
Yes	6	19.4
Previous AMS training		
No	23	74.2
Yes	8	25.8

Five major themes emerged from the study: commonly sold antimicrobials; knowledge on AMR and AMS; potential contributors to AMR (lack of knowledge, overuse, misuse, and abuse of antimicrobials such as non-adherence to dosage, self-medication, and purchase of drugs without a prescription); practices related to AMS (meetings, dispensing drugs and providing client advice, as well as record keeping and monitoring of drugs); and challenges to AMS (cost of healthcare, client satisfaction and retention, outdated guidelines, and business orientation of pharmacies).

### Commonly sold antimicrobials

From the IDIs, participants identified various antimicrobials being sold in private pharmacies to treat different conditions. Participants reported penicillins, cephalosporins, tetracyclines, and fluoroquinolones as the commonly dispensed classes of antimicrobials. The different antimicrobials prescribed included: azithromycin, Ampiclox^®^ (ampicillin and cloxacillin), amoxicillin, ciprofloxacin, Septrin^®^ (co-trimoxazole), metronidazole, Flucamox (amoxicillin and flucloxacillin), Augmentin (co-amoxiclav), cephalexin, doxycycline, and chloramphenicol.

According to the participants, these drugs were mainly being used to treat respiratory tract infections such as bronchitis and pneumonia; urinary tract infections especially among females; coughs; flu; diarrhoea among children; and other bacterial diseases. Participants also stated that there was an increased demand for macrolides, particularly azithromycin, due to the COVID-19 pandemic.

*“Of recent, it has been more of the macrolides, to be specific, azithromycin because clients heard it is used in the treatment of COVID-19. We have been receiving many clients who always come and ask for azithromycin.”* **Participant 27**

### Knowledge on AMR and AMS

A few participants, mainly those with advanced training, correctly defined AMR but many had some idea of what it is. Some participants related AMR to continued use of antimicrobials beyond the prescribed duration due to a client’s inability to recover, while others stated that it was treating antimicrobial infections without achieving the desired outcomes which consequently led to the change of prescriptions. In addition, many participants were able to identify the consequences of AMR among patients. These consequences included increased hospital visits and hospitalization, as well as increased treatment costs due to more expensive forms of treatment. However, there was a misconception about where the resistance was occurring. Many of the participants stated that the resistance was developed by the patient’s body after prolonged use of antimicrobials and not by the disease-causing microorganisms.

*“Antimicrobial resistance occurs when either a person has taken or used a drug for quite a long time and the body has created a mechanism of resisting the efficacy of that drug to treat that disease*.” **Participant 22**

In contrast, very few participants had ever heard of the terminology AMS. In most cases, participants understood what the word antimicrobial meant but were not familiar with the term stewardship. As such, many of the participants did not know how to define AMS. Upon explanation from the RAs, some of the participants were able to link AMS to the collective responsibility to control the misuse of drugs, monitoring to allow rational use of drugs, and any practices carried out to prevent AMR.

*“I am not very familiar with the term antimicrobial stewardship. I know what antimicrobial means but for stewardship, I really do not know. Maybe if you explain it to me.”* **Participant 04**

### Potential contributors to AMR

When participants were asked to identify factors contributing to AMR, they stated lack of knowledge, overuse, misuse, and abuse of antimicrobials such as non-adherence to dosage, self-medication, and purchase of drugs without prescriptions. Some of the participants felt that health workers had limited knowledge on what AMR was, its causes and consequences. The pharmacy staff stated that limited knowledge on AMR among health workers was shown by polypharmacy, irrational and prolonged prescription doses, and non-specific prescriptions as demonstrated by clients upon their visits to pharmacies.

*“There is a lack of knowledge on antimicrobial resistance among health professionals. On the professional side, there is what we call polypharmacy. This is when too many antibiotics are given to a patient. Maybe you needed one or two to kill the microorganisms but you find the health worker has prescribed three or four drugs to the same client.”* **Participant 17**

On the community side, participants reported that many of the clients who went to the pharmacies were not knowledgeable about AMR, hence unaware of the implications of misuse and abuse of drugs. As such, many participants stated that their clients usually used antibiotics whenever and however they wanted, thereby fuelling non-adherence and self-medication. In addition, some participants mentioned that many clients were used to taking incomplete doses and discontinuing the drugs prematurely. Reasons for discontinuation of drugs included when one felt better, dislike for medicines, and preservation for future use.

*“Lack of knowledge because they* [community members] *come and think that they know a lot about the drugs. Some even say ‘don’t give me that drug, it is for cough’ because they are used to it whenever they get cough. Other people do not complete the dosage provided when they feel better especially when they dislike taking drugs. So, when you look at the main cause of the problem, it is lack of knowledge.” ****Participant 18***

Whereas only a few of the participants reported the sale of antimicrobials for animal use in their pharmacies, it was noted that some of their clients requested human medication for animals under falsification. For example, some clients bought human drugs and used them on their animals. This was noted to be another potential contributor to AMR.

*“What could be causing AMR in the community mostly, is how people are using antimicrobials. You find that patients can even lie, and instead of buying medicines for themselves, they buy and use them to treat their animals or birds. So in that way, these bacteria get exposed to the antibiotics and develop a mechanism of resistance. That’s one of the major causes of antimicrobial resistance.”* **Participant 4**

### Practices related to AMS

A number of sub-themes were identified under the theme of practices related to AMS. These sub-themes included: meetings; dispensing drugs and providing client advice; as well as record keeping and monitoring of drugs.

#### Meetings

Although none of the pharmacies had a specific committee responsible for AMS, most of the participants reported having meetings where they discussed general issues arising from their day-to-day activities including AMS. These gatherings were either early morning physical meetings or via *WhatsApp* whose frequency was either daily, weekly or monthly. Particularly regarding AMS, participants reported discussion of new guidelines, treatments and drugs on the market, different ineffective antibiotics due to resistance, and client experiences regarding AMR. It was noted that although some of these meetings were not official, they always provided key information on AMS concerns at the pharmacies.

*“Yes, we have meetings now and again. We hold them with the owner of the pharmacy. We discuss many things in these meetings including the effectiveness of drugs. It might not be a real AMS committee but we learn a lot from these meetings*.” **Participant 17**

### Dispensing drugs and providing client advice

Participants stated that drugs were dispensed in three situations: with a health professional’s prescription; after assessment or explanation of the client’s symptoms within the pharmacy; and on self-prescription. For the latter two situations, antimicrobials are sold without prescription. According to the participants, prescriptions were mainly from neighbouring health facilities, personal and family doctors, and refills of previous prescriptions for chronic illnesses such as hypertension and diabetes. However, many of the participants reported that the larger percentage of clients they served had no prescriptions but generally requested antimicrobials based on past illness experience, previous prescriptions or medical consultations, as well as after seeking advice and suggestions from friends, family, and internet sources.

*“Many clients generally have no prescriptions. They either have a prescription from a friend, or others get it from their own experience. For example, some say ‘I used this drug and it worked for me so please give it to me again’. Others say they want that very treatment because previously they suffered from the same condition and used that medication which worked well.”* **Participant 13**

Among clients with prescriptions, advice was given based on whether the prescription was from a medical health professional or a non-medical professional, such as friends and family. Usually, prescriptions from medical professionals were not scrutinized unless the pharmacy staff thought the prescriber had given a wrong prescription for example due to polypharmacy. In such cases, the participants, either did clerkship and then recommended the right antimicrobial to the client or called the respective prescriber where possible to clarify any queries including change of prescriptions.

*“If it is possible, we engage with the prescribers and if it is not possible, we explain to the client what we know. So sometimes the clients go back and speak to their prescribers or they take the advice we give them.”* **Participant 19**

In cases where clients showed up without prescriptions, the participants reported taking client history and doing clerkship before they advised and prescribed antimicrobials. After discussion with clients, they would agree on the best treatment option, taking client financial capability into consideration. Advice on antimicrobial use was given to clients through counselling and health education sessions on adherence and compliance to antimicrobials, taking full doses of antimicrobials, prescribing drugs according to clients’ signs and symptoms, as well as requesting clients to go for medical check-ups or diagnostic tests to direct the type of antimicrobial to be used.

*“Yes, we do advise our clients. For most antimicrobials, we don’t sell quarter or half doses. Most times, we encourage clients to buy the whole dose and if they are not buying a whole dose because of money issues, we give them a variety of cheaper but full-dose antimicrobial brands rather than giving them a quarter dose because most don’t come back to buy the remaining dosage.”* **Participant 19**

When participants were asked to identify commonly used sources of advice during their practice, some pharmacy staff mentioned the use of the Uganda Clinical Guidelines (UCG), British National Formulary, online apps such as Medscape, and the internet, particularly Google. Some participants mentioned asking their work colleagues, especially pharmacists for advice whenever they were not sure of the prescriptions to give clients or patient self-care advice. Other participants said that they usually referred to the drug leaflets whenever they needed advice on how to prescribe antimicrobials especially for new drugs.

*“What I usually do is I use the manufacturers’ advice. When I get a new antibiotic brand, I first read the leaflets or I google using my phone, and even when we sit as a group, we discuss the new drugs. Every drug has a leaflet with side effects, and drug interactions and I think everything is always there, even the mode of administration.”* **Participant 5**

#### Record keeping and monitoring of drugs

Participants reported that their pharmacies had received a prescription book from NDA where they were recording the different antimicrobials dispensed. However, a few participants alluded that NDA had not given them new books in 2021 and 2022 therefore they had stopped taking records of the drugs prescribed. Other participants disclosed that NDA had not collected these prescription books for a long time hence record keeping was not a priority for their pharmacy, as no one asked for the records. As such, only a few of the pharmacies had been keeping updated records of the drugs dispensed. Many of the participants reported that their pharmacies were monitoring the different drugs dispensed mainly for accountability, stock taking (stock-outs and stock-ins), drug expiry dates, and sales forecasts. Other pharmacies kept records to restock drugs depending on demand within the community.

*“Yes, we used to* [keep records] *but this year we haven’t. I don’t know what happened because NDA which collects that data on antimicrobials and other drugs stopped coming. We do not know if they will resume.”* **Participant 19**

### Challenges to AMS

Regarding challenges to AMS practices, four sub-themes emerged: cost of healthcare; client satisfaction and retention; outdated AMS guidelines; and business-orientation of pharmacies.

#### Cost of healthcare

According to the participants, the high cost of healthcare was a major contributing factor to improper practices related to AMS. This is because many of the clients visited pharmacies without prescriptions, as they were avoiding the high consultation fees at private health facilities due to lack of money. Other participants highlighted that some clients also had limited funds therefore were unable to procure prescribed antimicrobials in full doses as stated by the healthcare professionals. Consequently, this led to self-medication and the purchase of incomplete doses.

*“Yes, people are poor. People nowadays are fearing to go to see health workers because they cannot afford the consultation fees and all those other related costs like diagnostic tests hence they prefer self-medicating.”* **Participant 27**

#### Client satisfaction and retention

Another challenge to AMS mentioned by the participants was client satisfaction and retention. Some participants mentioned that they needed to make sure their clients were satisfied with their services. In most cases, this meant pleasing them by giving them the antimicrobials requested, even if they were not appropriate, for example, when not backed up by any medical opinion or advice. Participants also reported that some of their clients had a fixed mindset of a particular type of drug either from the internet, a friend or previous experience, which made it hard to give them advice. Participants further stressed that such clients would usually expect the pharmacy staff to serve them their desired drug choice without any symptom assessment, previous medical history, self-care advice, or counselling on drug usage. Client satisfaction was closely linked to maintenance and expansion of the pharmacy client base, resulting in good sales. As such, the choice of antimicrobials given to the clients was influenced by the client to a certain extent. Other participants highlighted that failure to serve such clients with their desired drugs was not good for business especially if they did not recover.

*“But majority of our clients claim to know what they want hence you cannot tell them otherwise. The moment you change their choice and they don’t improve, it is a bad situation and because of that risk, we just give them what they want. So at times, you are forced to give a drug even when not ideal because of demand.”* **Participant 13**

#### Outdated guidelines

According to the participants, most of the AMS guidelines in Uganda were outdated and therefore not reliable. For example, some participants expressed concerns about the current UCG which had not been updated since 2016, yet some of the treatment options in these guidelines were no longer effective. Other participants mentioned that the paper-based format of guidelines, which had many pages, made it difficult for them to read the information as one had to flip through several pages of the document. This necessitated some of them to explore other sources of information such as the internet.

*“I would need to get information from a platform that is updated like google. I trust online sources more because these books like the Uganda Clinical Guidelines don’t have current drugs. I would want a system with up-to-date information.”* **Participant 5**

#### Business-orientation of pharmacies

Many of the participants felt that the implementation of AMS activities was difficult, as most pharmacies were in the business for profit. Some participants also emphasized this by stating that in Uganda, it was not common for a client to be denied an antibiotic at the point of service especially if the client had money to purchase the drug. This was because most pharmacies were more concerned with the number of clients and sales made each day. Some participants felt that the type of drug that was prescribed sometimes depended on the profits made and not on the patient’s illness, while others felt that some clinicians intentionally prescribed many drugs to increase their sales.

*“Business-oriented mindset is too common in most private pharmacies because they need to maximize profits to make a lot of money. Pharmacy staff might say these drugs work but yet they do not. They always want to prescribe something which is more expensive yet there are some drugs that are not expensive that might do better than the expensive one.”* **Participant 10**

## Discussion

Our results present the situation of AMS in private pharmacy settings in a typical LMIC which should contribute to the much needed evidence to reduce AMR. Efforts to optimize the rational use of antimicrobials at different levels of the healthcare system have been emphasized [40]. However, private pharmacies have been left behind in many LMICs such as Uganda even when they are first point of contact to a large population [41]. Despite poor knowledge of AMR and AMS, and inadequate prioritization of AMS in the private pharmacies, our study findings show that these facilities have the potential to contribute to Uganda’s fight against AMR. Indeed, private pharmacies are easily accessible and in a unique position to provide advice and raise awareness among the public provided they are motivated and equipped with adequate knowledge on AMR/AMS. Deliberate efforts to implement more AMS programmes focussing on private pharmacies could be a starting point.

Similar to our study findings, commonly sold antimicrobials have also been reported by other studies [19, 21, 22, 42–45] and were mainly used to treat urinary tract infections and respiratory tract infections. It is also important to note that the commonly sold antimicrobials in this study such as penicillin, tetracyclines, amoxicillin, cloxacillin, chloramphenicol, and ciprofloxacin have already been implicated in the accelerated emergence of AMR in previous studies [8, 45, 46]. Findings from our study indicate an increased demand for azithromycin. This finding can be linked to the fact that the study was carried out in 2022 when the country was still grappling with COVID-19 and azithromycin was used as a treatment for the disease. Indeed, similar findings showing increased demand for azithromycin have been reported in Saudi Arabia [47], Kenya [48], Egypt [49], and India [50] during the COVID-19 pandemic. Since then, it has been found that azithromycin did not improve survival or any other prespecified clinical outcomes in patients with COVID-19 [45, 51]. However, the unintended impact(s) of increased antibiotic use during the pandemic needs to be further explored.

Knowledge of the terminologies AMR and AMS in our study was lacking as many pharmacy staff did not know how to clearly define them. Similarly, a study on knowledge and perceptions on AMR and AMS among 60 staff at a national cancer referral centre in Uganda also reported that respondents had heard of the term AMR but not AMS [52]. A possible reason for this may be because most of the participants were of low professional training, that is certificate (38.7%) and diploma (58.1%) holders, and the majority (74.2%) had not received any AMS training. AMR in Uganda is still a silent public health challenge that is mainly addressed at tertiary and regional hospitals [53, 54]. Indeed the expanded roles of pharmacists supporting AMS interventions has been well documented but necessitate increased capacity and investment in this workforce [55, 56]. In this study, only one pharmacist was interviewed which indicates the need for AMS trainings to be extend to other health allied institutions that train lower pharmacy based (and other) cadres. Previous literature in Uganda shows low occupancy and physical presence of pharmacists at workstations especially in private pharmacies [19, 57]. Possible reasons for pharmacists’ low presence at private pharmacies include the inability of pharmacy owners to remunerate them sufficiently, a low pharmacist-to-population ratio, dual practice, and stress due to workload [57–59]. Further research is needed to quantify the implications of low cadre staffing in private pharmacies on AMS and AMR.

Participants believed that both health workers working in the pharmacies and community members served had little or no knowledge on AMR and its consequences to modern medicine. Our finding differs from studies done in Zambia [60], Thailand [61], and Sudan [62] where the private pharmacy staff were knowledgeable about AMR. These differences could have resulted from the level of education of the participants, presence of AMS programmes, and type of study (qualitative versus quantitative). The notion that community members have inadequate knowledge on AMR is similar to studies in Tanzania [63], rural Mozambique [64], and the Democratic Republic of Congo [65]. These studies showed that participants were unaware of the risks associated with improper antibiotic use. Some of the participants in our study stated that AMR develops as a result of the patient’s body being resistant to drugs. This misconception has been documented in studies carried out in European and African countries where patients viewed resistance as a property of the human body rather than of infecting organisms [64, 66, 67]. Misconceptions by healthworkers confound miscommunications on AMR and as such, our findings show the urgency to provide further education and training to frontline healthworkers in all settings and cadre, increase awareness campaigns on AMR and its consequences, and the immediate need for enhanced AMS in private pharmacies. Pharmacy affiliated health professionals are well placed to support AMS interventions if they possess the capability (knowledge of medicines with curricula to include AMR and AMS in training), opportunity (contact with prescribers and patients), and motivation (Pharmaceutical Society of Uganda’s ethical standards for good professional practice) [23].

From the study findings, dispensing of drugs without prescriptions was the norm as observed elsewhere in Eritrea [68], Tanzania [26], China [69], and Zambia [60]. Indeed, over 50% of drugs in Uganda are sold over the counter [18, 70]. The misuse and overuse of antimicrobials, which is inclusive of self-medication and the dispensing of drugs without a prescription, is a leading driver of AMR in humans [71, 72]. In our study, private pharmacy operations included these practices despite prohibitions of over the counter dispensing of antimicrobials from NDA. Our findings could have been as a result of the business orientation of pharmacies, client satisfaction and retention. Similarly to our study, client satisfaction and demand from patients are recurring factors in dispensing drugs without prescription among private pharmacies elsewhere [30, 62, 68, 73, 74]. Patient expectations and demand for antibiotics has also been expressed among lower levels of the health system including the community [75]. Conversely, a study on the impact of antibiotic prescribing for viral upper respiratory tract infections showed that patient satisfaction was not associated with receiving antibiotics [76]. As such, this study showed that pharmacy staff can meet patient satisfaction without unnecessary dispensing of antimicrobials. This calls for continuous professional education with tools such as the TARGET Antibiotic Checklist which has an emphasis on good antimicrobial prescribing practices and self-care advice for private pharmacies [77].

In our study, participants felt that the implementation of AMS activities was challenging due to the business-oriented nature of the pharmacies. A recent study carried out showed that at least half of the pharmacies had a mean operating profit margin greater than 18% [78]. Concerns about pharmacy owners refusing their employees to allow clients to leave the pharmacy premises without any dispensed medication, even when the clients did not need antimicrobials, have been documented [79]. Turning away clients is believed to cause a direct reduction in sales and profits for pharmacies and possibly lead to bankruptcy [79, 80]. Educating pharmacy staff on AMS may not have the desired impact if the number of sales made is used as part of their appraisal. This situation consequently puts pharmacy staff in a difficult situation regarding their obligation to improve health and the fear of being laid off due to low sales. To avoid such a scenario, we recommend increasing awareness on the impact of AMR for both private pharmacy employers and employees. In addition, more strict regulation and enforcement by the NDA regarding recommended practice in these settings is needed.

Our findings showed laxity by the pharmacy regulatory body as participants mentioned the use of outdated guidelines and the failure of NDA to distribute and collect drug prescription books. Regulation of private pharmacies is a global challenge that can negatively impact on AMR if poor practices are not addressed [81–83]. Updating of the UCG should occur regularly as the guidelines currently in use were developed in 2016 [84]. It is worth noting that at the time of the study, the UCG guidelines were under review by the MOH. As of 2018, the NDA no longer printed prototype prescription books for sale, and all pharmacies were to put in place their own system for recording prescriptions dispensed [85]. As part of this study, none of the participants mentioned this change. Therefore, we believe that the pharmacy staff were not aware of the new regulation. However, it should be noted that failure to receive a prescription book from NDA and the absence of pharmacy inspection in relation to existing books were linked to reduced prioritization for record keeping. A study on the compliance of private pharmacies in Uganda to control prescription drug regulation found poor (23%) adherence to stock control requirements [82]. Insufficient record keeping results in poor monitoring of drugs which slows down surveillance efforts on antimicrobial use as it undermines accountability [86]. Regulation activities in private pharmacies could include a well-recognized structure of inspection and routine support supervision visits from NDA. However, such inspections should not be characterized by impromptu raids that are harsh and instil fear among pharmacy staff, but rather create space for continuous review of needs, knowledge, and resources among pharmacies [87].

Many pharmacy staff mentioned giving advice and recommending drugs to clients after ascertaining patient history and clerkship for clients without prescriptions. However, due to increased morbidity and mortality from resistant infectious diseases, clinical diagnosis followed by laboratory diagnosis before the prescription of drugs is strongly encouraged including in private health facility settings [62, 88, 89]. LMICs such as Uganda could learn from high-income countries where dispensing of drugs without prescriptions is minimal [90]. However, the regulation of antimicrobials in Uganda should be in such a way that it does not undo the gains of accessibility to medicines especially to vulnerable and hard-to-reach populations [91]. Furthermore, our study findings revealed pharmacy staff had meetings on general issues arising from their day-to-day activities including some related to prescribing antimicrobials. In addition, participants stated use of guidelines, online apps, reading of drug leaflets, and acquiring advice from either colleagues or pharmacist supervisors when they needed guidance. The desire of private pharmacy staff to acquire advice from colleagues and other sources could be used to enhance their knowledge to become antimicrobial guardians. Through CwPAMS, a free mobile application which contains the UCG, IPC guidelines, as well as information on AMS and AMR has been launched which could be utilized among private pharmacies [36].

The use of human medicines for animal use is a potential contributor to escalating levels of AMR which is echoed by studies in Cambodia [92], India [93], Uganda [37], and Ethiopia [94]. In our study amoxicillin, chloramphenicol, and metronidazole were among the most commonly dispensed antibiotics for animal use. A cross-country study between Uganda, Tanzania and India reported the above antibiotics among the most commonly used human antibiotics in animals [95]. Cross-use of antibiotics from humans to animals contributes to the development and spread of drug-resistant microorgansims as they can enter food chains and be spread through the environment and waterways. To increase end-user knowledge on AMR and AMS, contextualized community engagement opportunities using a One Health approach to facilitate behaviour change has been suggested [96].

We observed some limitations in our study. First of all, the RAs felt that some of the pharmacy staff were withholding information during the interviews. Indeed, some private pharmacies that were approached declined to take part in the study out of fear that the RAs could have been sent by the regulatory body. To minimize this concern, the RAs were coached on how to create a safe space for participants to fully express themselves, as well as clearly explain the purpose of the study. In addition, the RAs emphasized that they were not working for any regulatory authorities and all data were to be anonymized. The RAs might also have caused unintentional bias among the participants when explaining AMR and AMS in lay terms. However, a clear understanding of these terms was of importance in answering the proceeding questions in the interview guide. Our study was conducted in an urban setting hence the findings may not be generalizable to other settings in the country. Nevertheless, this is among the first studies on AMS in private pharmacies in central Uganda which can inform future interventions to reduce AMR in the country.

## Conclusion

The study revealed that most of the participants had limited knowledge on AMR and AMS, as well as reported undesirable practices related to dispensing of antimicrobials such as being profit oriented and prescribing based on patient demands. This highlights the need for further training of private pharmacy staff to improve their understanding of AMS and its importance in preventing the emergence and spread of AMR. This study also raised the need for greater public awareness about AMR as the sale of antimicrobials was driven by consumers’ demands. Private pharmacies can contribute to Uganda’s fight against AMR if motivated and equipped with adequate knowledge to enhance their practices related to AMS. It is also important that private pharmacies provide a unified advisory role for the public with regard to the provision and use of antimicrobials. In addition, strengthened regulation and enforcement among private pharmacies by regulatory authorities is crucial for enhanced AMS.

## Data Availability

Data from study are available from the corresponding author on reasonable request.

## Abbreviations

AMR: Antimicrobial resistance
AMS: Antimicrobial stewardship
IDI: In-depth interview
IPC: Infection prevention and control
LMIC: Low- and middle-income country
MOH: Ministry of Health
NDA: National Drug Authority
RA: Research assistant
WHO: World Health Organization
